# Glucocorticoid Induced Cerebellar Toxicity in the Developing Neonate: Implications for Glucocorticoid Therapy during Bronchopulmonary Dysplasia

**DOI:** 10.3390/cells3010036

**Published:** 2014-01-08

**Authors:** Kevin K. Noguchi

**Affiliations:** Department of Psychiatry, School of Medicine, Washington University in St. Louis, 660 South Euclid, Box #8134, St. Louis, MO 63110, USA; E-Mail: noguchik@psychiatry.wustl.edu; Tel.: +1-314-362-7007; Fax: +1-314-362-2474

**Keywords:** bronchopulmonary dysplasia, dexamethasone, betamethasone, hydrocortisone, cerebellum, 11β-Hydroxysteroid Dehydrogenase Type II, cerebellum, apoptosis, external granule layer, neural progenitor cell

## Abstract

Prematurely born infants commonly suffer respiratory dysfunction due to the immature state of their lungs. As a result, clinicians often administer glucocorticoid (GC) therapy to accelerate lung maturation and reduce inflammation. Unfortunately, several studies have found GC therapy can also produce neuromotor/cognitive deficits and selectively stunt the cerebellum. However, despite its continued use, relatively little is known about how exposure to this hormone might produce neurodevelopmental deficits. In this review, we use rodent and human research to provide evidence that GC therapy may disrupt cerebellar development through the rapid induction of apoptosis in the cerebellar external granule layer (EGL). The EGL is a transient proliferative region responsible for the production of over 90% of the neurons in the cerebellum. During normal development, endogenous GC stimulation is thought to selectively signal the elimination of the EGL once production of new neurons is complete. As a result, GC therapy may precociously eliminate the EGL before it can produce enough neurons for normal cerebellar function. It is hoped that this review may provide information for future clinical research in addition to translational guidance for the safer use of GC therapy.

## 1. Introduction

While the definition of bronchopulmonary dysplasia can vary, it is often described as oxygen dependency in prematurely born infants that extends to 34 weeks corrected age [1]. Prematurely born infants often suffer from bronchopulmonary dysplasia due to the immature state of their lungs. As a result, glucocorticoids (GCs) are administered to accelerate lung maturation and reduce inflammation. Although GC therapy has been used for over 50 years, there has been an increasing concern that their use may iatrogenically produce neurodevelopmental deficits. Historically, much of this interest began in 1994 when the National Institutes of Health issued a Consensus Statement which strongly advocated the use of prenatal GC therapy for the prevention of bronchopulmonary dysplasia [2]. The medical community responded vigorously, eventually leading to the widespread adoption of prenatal GC therapy in addition to the use of postnatal and/or more chronic GC dosing regimens that the report did not recommend. This trend of increased GC use continued until it was found individuals exposed to postnatal GC therapy exhibited permanent neuromotor/cognitive deficits [3,4,5] and, later, selective cerebellar stunting [6,7]. Due to escalating concerns regarding GC’s iatrogenic potential, the National Institutes of Health released a second Consensus Statement, which maintained that antenatal GC therapy should still be used but recommended against the chronic and/or postnatal drug regimens that were becoming more common [8]. The following year, the American Academy of Pediatrics recommended against the systemic use of postnatal GC therapy outside of limited double blind placebo controlled studies [9]. However, in 2010, this was changed to the recommendation that the clinician balance the potential adverse and beneficial effects when administering postnatal GC therapy [10]. Currently, approximately 19% of very low birth weight infants receive this treatment, however, its use remains highly individual/institution specific suggesting no clear guidelines for its use [11,12]. Yet, despite this continued use, relatively little is known about how this treatment might produce neuromotor/cognitive deficits and cerebellar stunting, which may provide further guidance for its use. 

**Figure 1:**
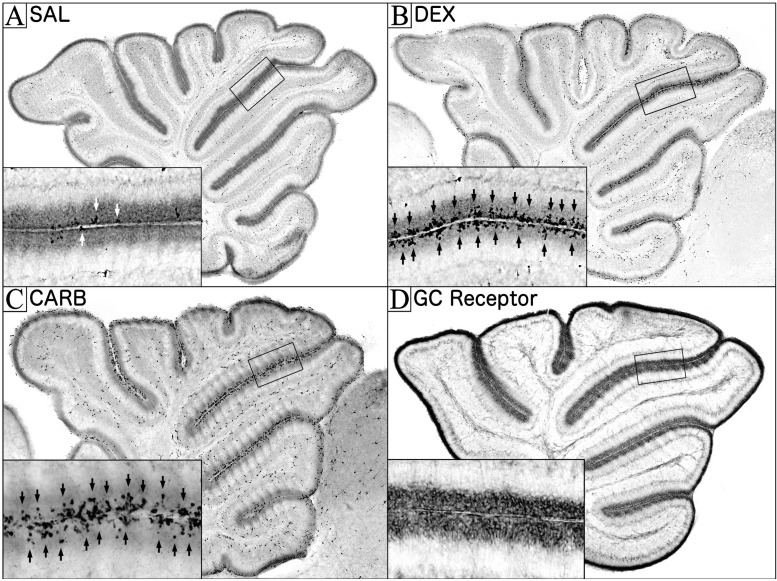
Glucocorticoid receptor stimulation in the neonatal rodent cerebellum. (**A**–**C**) Immunolabeling in the midsaggittal mouse cerebellum with the apoptotic marker activated caspase-3, six hours after saline (SAL), 3.0 mg/kg dexamethasone (DEX), or 100 mg/kg carbenoxolone (CARB). (**A**) SAL treated animals exhibited low amounts of physiological apoptotic death (white arrows), whereas (**B**) DEX treated animals exhibited dramatic increases in apoptosis (black arrows) throughout the EGL; (**D**) Simply, inhibiting HSD2 (an enzyme which metabolizes and protects against certain GCs) with 100 mg/kg carbenoxolone (CARB) rapidly increases NPC apoptosis in the EGL (black arrows). This suggests that when HSD2 is present, it is constantly preventing endogenous GC stimulation from producing EGL apoptosis. (**C**) Immunolabeling with the GC receptor antibody M20 reveals GC receptors are densely located in the EGL of the PND7 neonatal mouse. Insets reflect magnified views of boxed regions.

The nervous system is the most vulnerable system to developmental injury due to the complex ontogenic processes that occur over multiple stages [13]. Therefore, it is not surprising that the ability of GCs to stunt brain development in the rodent was known [14] even before Buckingham *et al.* [15] first suggested this steroid might accelerate lung development. Since then, multiple studies have established that GCs can dramatically effect rodent cerebellar development by decreasing proliferation [16,17,18]. Unfortunately, despite these advances, it was still not clear how this decrease in proliferation is occurring or why such vulnerability would exist to begin with. As a result, we examined the possible toxic effects of neonatal GC exposure in the rodent. We discovered that a single clinically relevant GC injection produced a rapid (within four hours), selective, and dramatic increase in the number of degenerating neural progenitor cells (NPCs) in a proliferative layer called the external granuler layer (EGL) of the developing cerebellum (Figure 1A–B) [19,20]. Further research suggested endogenous GC stimulation was carefully orchestrated to naturally signal increased NPC death, as the EGL is naturally eliminated from the cerebellum [19]. Therefore, the precocious stimulation caused by GC therapy may cause the premature deletion of this important proliferative region, leading to cerebellar stunting. In this paper we will review the role GCs play in cerebellar development and explain how the premature exposure to this hormone may iatrogenically produce disruptions in cerebellar formation and behavior. 

## 2. Glucocorticoid Induced Neural Progenitor Cell Death in the Cerebellum

To examine how GCs may produce neurodevelopmental deficits, we exposed neonatal mouse pups to GCs and screened the entire brain for apoptosis (programmed cell death). We found that single exposure to multiple types of GCs all induced selective NPC apoptosis in the EGL within 4–6 h [19,20]. Similar to clinical research, we also found this exposure caused permanent neuromotor deficits and selective cerebellar stunting [19,20,21]. The most commonly used drug for GC therapy (dexamethasone) was able to increase NPC apoptosis at doses of 0.03 mg/kg and higher. This is approximately 10 times lower than the dose commonly administered to prematurely born infants [19]. While comparing doses between rodent and human can be difficult, the prevailing view is that humans metabolize dexamethasone and other GCs more slowly than other species [22,23]. As a result, the doses we found to be toxic in the mouse would be expected to be even more potent in the human. Before continuing, in order to fully explain the how this toxicity may have clinical relevance to GC therapy, it will be important to elucidate the important roles apoptosis and the EGL play in normal cerebellar development. 

### 2.1. The EGL and Its Role in Cerebellar Development

**Figure 2:**
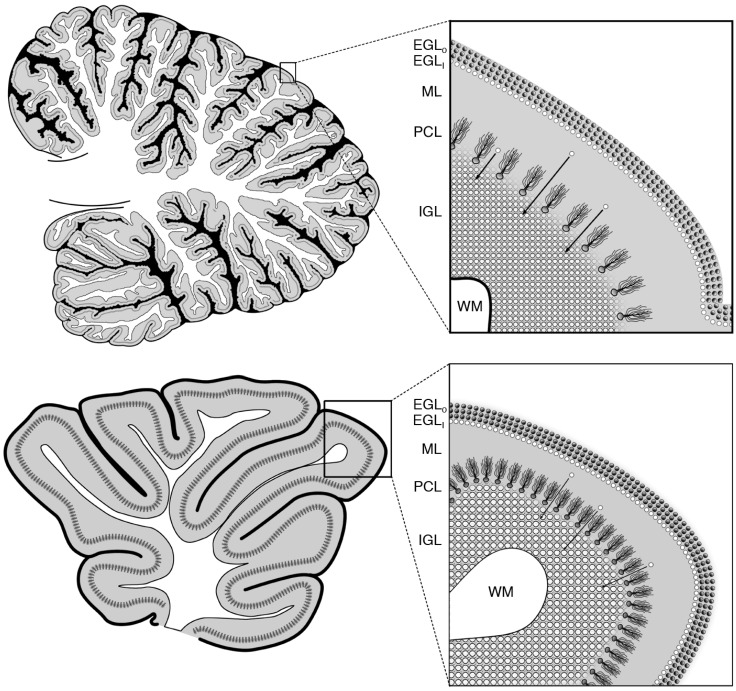
Midsaggittal diagram of the human and rodent cerebellum and its constituent layers during perinatal development. The gyrencephalic nature of human cerebellum (TOP) leads to more gyri and sulci than the more lissencephalic rodent cerebellum (BOTTOM). Despite these differences, the human and rodent cerebella maintain the same basic cortical cytoarchitecture. In both species, the outermost layer of the cerebellum is the transient external granule layer (EGL), which is composed of an outer germinal matrix populated by neural progenitor cells (EGL_O_; dark spheres) and an inner layer (EGL_I_; white spheres) where newly formed granule cell neurons congregate and mature. After a short time, these granule cells then migrate (white spheres with arrows) past the molecular (ML) and Purkinje cell layers (PCL) before becoming incorporated into the internal granule layer (IGL), which lies superficial to the cerebellar white matter (WM). After birth, the EGL begins to disappear as its job of producing new neurons ends, and the cerebellum transitions into a more adult state. Insets (right) reflect magnified views of boxed regions (left).

In both humans and rodents, the production of neurons occurs in specialized germinal matrices that proliferate through cell division to produce new NPCs and/or neurons. Almost all of them are transient structures which, after their job of producing new neurons is over, are naturally eliminated from the brain. One of the last of these transient proliferative regions is the EGL, which occupies the outermost layer of the developing cerebellum (Figure 2). The EGL is composed of two sublayers consisting of an outer germinal matrix containing NPCs that proliferate to form new neurons/progenitor cells and an inner portion that temporarily harbors newly formed granule cell neurons (Figure 2). After these new granule cells mature in the inner EGL, they migrate past the molecular and Purkinje cell layers and eventually populate the internal granule layer. As a result, the EGL produces neurons solely for the internal granule cell layer with all other neuronal cell types being produced by other proliferative layers earlier on in development [24]. The amount of neurogenesis occurring in the EGL is quite substantial, producing over 90% of the neurons in the cerebellum. As the cerebellum actually contains more neurons than the cerebrum, EGL produces over half (with some estimating over 80%) the neurons in the entire brain [25,26,27]. 

The cerebellum of the rodent and human show a remarkable homology. This includes similarities in their characteristic cerebellar layers, signaling molecules, constituent neuronal subtypes, their synaptic connectivity, and the use of the EGL as a germinal matrix for granule cell neurogenesis [28]. The major difference between species is the degree of gyrencephalization (Figure 2) and that the rodent cerebellum in a more immature developmental state at parturition. During normal mouse development, the EGL is a thin layer of progenitors at birth, but rapidly expands to maximal thickness within a few days. Around two weeks of age, neurogenesis naturally ceases and this region permanently disappears from the cerebellum [29,30]. Alternatively, during normal human development, the EGL begins to expand during the second trimester of gestation, reaches maximal thickness prenatally, and finally begins disappearing soon after birth [20,31]. Based on this information, GC therapy is typically given while the vast majority of cerebellar neurons are being produced but after almost all cerebral neurogenesis has ended [32]. Our estimates suggest a comparable human window of vulnerability for this GC induced cerebellar toxicity would correspond to 20 weeks in gestation to 6.5 weeks after birth [20]. 

### 2.2. Apoptosis and Development

At this point, it is worth noting that, while the logical concept of maturation is often associated with growth, normal development often involves the selective death of certain cells. In particular, it is broadly recognized that the body maintains an active process called apoptosis in which specific cells are purposefully deleted and undergo a predictable sequence of degenerative events [33]. As the apoptotic death of an NPC not only deletes that cell, but also eliminates every NPC/neuron which that cell would produce in the future, it can have a magnified effect when compared to neuronal apoptosis. As a result, when NPC apoptosis is reduced, the offspring display dramatically enlarged brain malformations [34,35,36] but, when NPC apoptosis is increased, the resulting brain showed dramatic decreases in brain size [37,38,39,40]. As a result, due to the exponential growth of this cell population, the dysregulation of NPC apoptosis can drastically affect the final number of neurons produced in the adult. Ultimately, these studies have positioned the regulation of NPC apoptosis as a critical step in normal neurodevelopment and led to a great interest in finding novel signaling molecules, which can regulate this process [41,42,43,44,45]. Consistent with this idea, the EGL apoptosis produced by a single GC exposure can permanently reduce the number of cerebellar granule neurons by 18% [20]. As the internal granule layer neurons represent well over half the neurons in the brain [25,26,27], this means a single injection of dexamethasone, from a purely numerical standpoint, reduced the total number of neurons in the brain by at least 9%. 

## 3. The Role of Glucocorticoids in Cerebellar Neurodevelopment

The curious ability of GCs to induce NPC apoptosis logically leads to one final question. Why would this selective vulnerability exist to begin with? Interestingly, several lines of evidence suggest that GCs may be an endogenous apoptotic signal leading to the removal of the EGL once its done producing neurons. Such a finding would be consistent with the well-known ability of GCs to regulate apoptosis in several other organ systems [46,47] and act as a maturational signal which transitions numerous organs into a more mature state [48]. In this section, we will present evidence that GCs may provide a maturational signal to apoptotically remove the EGL, thereby transitioning the cerebellar cytoarchitecture to that of the adult [19,20]. 

### 3.1. GC Stimulation in the EGL is Carefully Regulated during Its Disappearance

If GC’s signal the removal of the EGL, the body would need to maintain intricate regional and temporal control over endogenous stimulation in order for the cerebellum to develop correctly. One of the first indications of this is GC receptor expression. Pavlik *et al.* [49] showed that the rodent neonatal cerebellum has GC receptor levels more than three times higher than any other region of the brain. Interestingly, these receptor levels increased as the EGL was expanding but decreased as the EGL was disappearing, suggesting a large portion were localized to the EGL. In order to determine what cerebellar sublayers contain these receptors, we performed GC receptor immunohistochemistry and discovered that the highest concentration, by far, was in the EGL (Figure 1D) [20].

A second way the body regulates GC stimulation is through endogenous GC release. After birth, there is a dramatic suppression of endogenous corticosterone (the rodent equivalent of cortisol) levels and a reduced ability to release this hormone in response to stressors [50,51]. This rodent “stress hyporesponsive period” begins before EGL NPCs become vulnerable to GC induced NPC apoptosis. After the neonate becomes susceptible to this toxicity, endogenous corticosterone levels only rise to adult levels around the same time the EGL decreases proliferation and begins to disappear [49,50,52,53,54]. In order to test what effect this rise might have on apoptosis, we increased corticosterone levels during the stress hyporesponsive period to levels normally seen in the adult. This treatment produced a significant increase in EGL apoptosis suggesting the natural surge of this hormone during maturation may help remove the EGL through GC induced apoptosis [19]. Conversely, we also inhibited endogenous GC stimulation by administering a GC antagonist and found this reduces physiological EGL apoptosis in neonatal pups [19]. Taken as a whole, this research suggests that the rise in endogenous GCs, as the stress hyporesponsive period ends, increases EGL apoptosis, thereby contributing to its elimination.

A final way the body can regulate endogenous GC stimulation is through the expression of the enzyme 11β-Hydroxysteroid Dehydrogenase Type II (HSD2), which selectively protects tissue from GC stimulation by rapidly inactivating endogenous GCs before they can stimulate GCs receptors [52,53]. This enzyme is thought to be involved in neurodevelopment due to its high expression throughout the rodent brain in early gestation [55]. Around mid-gestation, levels in the brain decrease rapidly until HSD2 is almost exclusively isolated to the EGL at birth. As the neonate ages, high HSD2 levels are maintained until disappearing as the EGL is naturally eliminated from the cerebellum [52]. In order to test whether this natural decrease in HSD2 might contribute to the disintegration of the EGL, this enzyme was inhibited in neonatal mouse pups and the cerebella were screened for apoptosis. The results revealed that simply inhibiting this enzyme during the stress hyporesponsive period dramatically increased NPC apoptosis within a few hours (Figure 1C) [19]. It was also found that this toxicity could be blocked by pretreatment with GC receptor antagonist suggesting it is produced through endogenous GC stimulation [19]. These results suggest that, during the stress hyporesponsive period, HSD2 is constantly protecting the EGL from apoptosis. Interestingly, the ability of HSD2 to protect the EGL may have important implications for perinatal GC therapy. This is because HSD2 is efficient at protecting against endogenous GCs, such as hydrocortisone (Note: when the endogenous GC cortisol is administered as a drug it is often called hydrocortisone), but has a very limited ability to protect against the most commonly used synthetic GCs (e.g., dexamethasone and betamethasone). As a result, many synthetic GCs bypass the natural protection afforded by HSD2 in the EGL. Consistent with this idea, equipotent doses of endogenous GCs produce less NPC apoptosis than dexamethasone or betamethasone [19]. It should be noted that, while the HSD2 enzyme is also regionally expressed in the lungs, it is not present in the alveoli, which contain surfactant producing cells (type II pneumocytes) that improve respiratory function [56]. As a result, using endogenous GCs, such as cortisol (*i.e.*, hydrocortisone), may allow HSD2 to selectively protect the EGL while allowing the therapeutic effects on lung maturation to occur. Consistent with this concept, recent research found increasing HSD2 expression in the EGL reduced GC induced EGL apoptosis [57].

Taken as a whole, these findings strongly suggest endogenous GC stimulation plays a maturational role by apoptotically eliminating the EGL once its job of producing neurons is over. Other research also supports this hypothesis. Firstly, neonatal injections of GCs leads to the premature removal of the EGL, decreases EGL proliferation, and decreases brain size [17,18,58,59]. Conversely, decreasing endogenous GC levels through adrenalectomy dramatically delays the normal disappearance of the EGL, increases EGL proliferation, and increases brain weight [60,61,62]. Finally, other research has found the EGL in wild-type mice disappears by PND16 but transgenic mice with impaired apoptosis display a robust mitotically active EGL with preserved migration of granule cells to the internal granule layer [34]. This shows apoptosis is critical for the normal elimination of the EGL despite any effects on proliferation, migration, and/or differentiation. 

### 3.2. Interactions between GC Stimulation and the Sonic Hedgehog Pathway

While it is clear GCs increase EGL apoptosis, it has recently been suggested that this steroid also interacts with the Sonic Hedgehog Pathway (ShhP) to effect cerebellar development. During EGL neurogenesis, Purkinje cells carefully regulate the number of granule cells so the ratio between the two is consistent [63]. In order to accomplish this, Purkinje cells release a soluble morphogen called the Sonic Hedgehog ligand, which diffuses to the EGL and stimulates NPC proliferation by activating the ShhP (a potent mitogenic signal) [64]. Importantly, Heine and Rowitch (2009 and 2011) recently found that both transgenic and pharmacologic stimulation of the ShhP increases HSD2 expression in the EGL [57,65], which can then protect against GC induced EGL apoptosis. This discovery makes logical sense in that, as the ShhP and proliferation naturally increases, the EGL would need to protect these cells from apoptosis through increased HSD2 expression. Ultimately, this suggests the ShhP serves a previously unknown function of regulating GC stimulation in the EGL through HSD2 protection. 

Heine and Rowitch (2009) also reported that one downstream effect of GC stimulation is the inhibition of the ShhP leading to reduced EGL proliferation independent of apoptosis. Unfortunately, in subsequent papers this same group has been unable to replicate this cell culture work *in vitro* [57] and an attempt to reproduce this effect *in vivo* has also failed [66]. It should also be noted that the *in vitro* work used dexamethasone (a synthetic GC) at concentrations between 40–160 uM [65], but the highest clinically relevant concentration is considered to be 1–10 uM [67,68]. Of further concern, two independent groups recently did large screens, looking for novel drugs that may affect the ShhP. Instead of finding GCs inhibit the ShhP, they found numerous GCs directly stimulated [69,70] or potentiated [71] the ShhP in EGL NPCs or other cell types. As this agonism showed dose dependent competition with Smoothened agonists (Smoothened is a critical component of the ShhP, which is the target of numerous drugs affecting ShhP stimulation), it is highly likely this effect occurs on an off-target site independent of the GC receptor. The GC concentration needed to affect the ShhP occur at levels 10 times higher than those needed to affect GC stimulation [71]. As a result, stimulation of the ShhP on EGL NPCs should be eliminated by the eventual apoptosis of that NPC. While it was found that a much smaller number of GCs could inhibit the ShhP, dexamethasone was found to increase ciliary accumulation in NIH 3T3 cells (a requirement for ShhP activation, which suggests that dexamethasone potentiates the ShhP) [71]. Taken as a whole, these findings strongly suggest that ShhP inhibition is not an inherent downstream effect of GC stimulation. As a result, while this may potentially have important implications for cerebellar development, further research is clearly needed. 

Heine and Rowitch (2009) also found different effects with acute *versus* chronic GC stimulation on EGL apoptosis. While they confirmed acute GC exposure produces EGL apoptosis, it was also reported that chronic exposure (*i.e.*, dexamethasone exposure once a day from postnatal days 0–7) produces no apoptosis when compared to chronic saline exposure [65]. Such a finding would be important in that it would suggest that acute and chronic GC exposure affect cerebellar development through fundamentally different mechanisms. Unfortunately, it has been pointed out that the apoptotic marker used in this study lasts less than 12 h after a cell becomes apoptotic and, as a result, did not quantify all apoptotic NPCs during the eight-day course of GC exposure. It was further shown that when a more appropriate experimental design is used chronic GC exposure does indeed produce EGL apoptosis [19]. As a result, it is still uncertain if GCs can directly affect NPC proliferation that is independent of NPC apoptosis. Of course, as one of the downstream effects of NPC apoptosis is decreased proliferation, distinguishing between these two effects is inherently difficult.

## 4. Does GC Stimulation Have Similar Cerebellar Effects in Humans?

Clinical research also suggests the cerebellum may be particularly susceptible to GC exposure. Correlational research has found that the most stunted brain region following postnatal GC therapy is the cerebellum, which exhibits a 20.6% decrease in volume on average [6]. A similar study found children exposed to GC therapy exhibited stunted cerebellar growth but had no measurable difference in the cerebrum [7], suggesting that this is a consistent finding. If this stunting leads to cerebellar dysfunction, it may also explain the neuromotor [72] and cognitive [72,73,74] deficits seen following GC therapy [3,4,5]. Certainly, the role of the cerebellum in balance and coordination has been established for quite some time [72]. However, research has also firmly established that preterm infants exposed to selective cerebellar hemorrhagic injury (which some believe disrupts cerebellar development by injuring EGL NPCs) display a high prevalence of cognitive and learning deficits [73,74]. These findings are supported by studies in adults and children with isolated cerebellar injury who display similar deficits [72].

If GC therapy is causing EGL toxicity in the human, this research may provide insight into improving drug treatment. As mentioned previously, HSD2 is effective at protecting against endogenous GCs but has a limited ability to metabolize the synthetic GCs most commonly used clinically (i.e., dexamethasone and betamethasone). As a result, the clinical use of these synthetic GCs bypasses the protective effect of HSD2 in the cerebellum [19,65]. While this may seem to propose a dilemma for the clinician, it may be possible to minimize these harmful effects by administering GCs HSD2 can metabolize [19,57]. Interestingly, several clinical studies have looked at whether endogenous GCs may be less harmful to prematurely born infants. It was reported hydrocortisone is effective at reducing oxygen dependency but does not produce the structural or neurodevelopmental deficits associated with postnatal dexamethasone exposure [75,76,77,78,79,80]. While this research seems promising, others have concluded hydrocortisone, while not harmful, is ineffective at treating respiratory dysfunction and should not be used [81]. Additionally, some have even reported hydrocortisone stunts the cerebellum [7]. As a result, there appears to be a strong interest in this area of research which is still evolving. 

It should also be noted that there are important differences one should consider when using hydrocortisone. Firstly, while dexamethasone and betamethasone have selective affinity for the GC receptor, hydrocortisone additionally stimulates mineralocorticoid receptors that may have other effects such as disturbing salt and water balances. Secondly, in the adult, 80%–90% of cortisol is bound in an inactive form by corticosteroid binding globulin (transcortin) [82]. As transcortin does not bind to most synthetic GCs it has limited pharmacokinetic effects on dexamethasone or betamethasone [82]. However, in the neonate and premature infant, there are extremely low physiological levels of this globulin at birth but these levels significantly rise with age [83,84,85]. As a result, transcortin’s ability to inactivate hydrocortisone may dynamically affect GC stimulation dependent on the amount of unbound transcortin at that particular age. Therefore, transcortin is an additional factor that needs to be considered when administering hydrocortisone.

**Figure 3:**
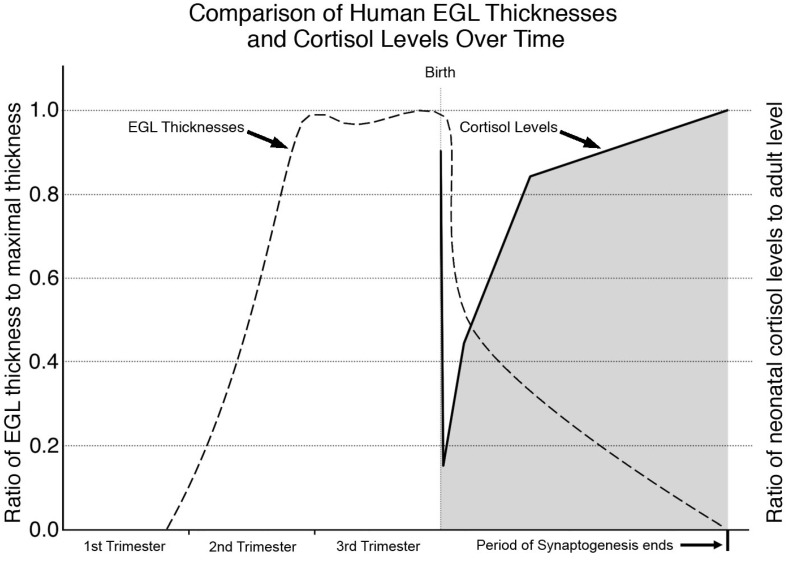
Comparison of human EGL thicknesses and cortisol levels during perinatal development. Graph depicting the thickness of the EGL expressed as a ratio to maximal thickness (dashed line no shading; left) compared to endogenous cortisol levels expressed as a ratio to the adult level (solid line grey shading; right) during perinatal human development. As endogenous cortisol levels naturally surge to adult levels, NPC apoptosis in the EGL increases as this proliferative layer’s thickness decreases rapidly, possibly reflecting cortisol induced apoptosis. Developmental time points are based on a full-term birth occurring at 37 weeks of pregnancy and the period of synaptogenesis ending two years after birth. Calculations of thickness and cortisol ratios are based on data from papers by Rakic & Sidman [31] and Sippell, Dörr, Bidlingmaier, & Knorr [84], respectively.

### Human Neurodevelopment and the Cerebellum

Next, one might ask if cortisol also plays a role in human cerebellar development. Unlike the rodent, the human EGL undergoes initial expansion in the prenatal womb (Figure 3) where the maternal, placental, and fetal environments combine to reduce cortisol exposure to the fetus [80] and EGL apoptosis is at a minimum [86]. As the pregnant body prepares for birth, there is a surge in maternal free cortisol levels a few hours before birth [87] that leads to fetal organ maturation in preparation for life outside of the womb [48]. After birth, there is a brief decline in cortisol levels until the neonatal adrenal gland begins producing its own cortisol, which eventually increases to levels comparable to that of the adult [84]. Similar to the rodent, around the same time we see this surge in cortisol levels, the EGL begins to disappear [31,84] (Figure 3) which is accompanied by a dramatic increase in EGL apoptosis that results in its eventual disappearance [86]. Therefore, as in the rodent, the specific timing of increased GC exposure in the human coincides with increased apoptosis and removal of the EGL. 

## 5. Conclusions

While GCs are best known for their role in adult stress, they serve a fundamentally different role during development by serving as a critical signal that transforms the constitution of multiple organs into a more mature state. For example, GCs signal the liver to increase glycogen storage, the thyroid to release hormones to control metabolism, and the lungs to stimulate surfactant release and initiate alveolar differentiation (which is why they are used to treat bronchopulmonary dysplasia) [48]. These maturational effects also extend to the brain where GCs can effect hippocampal proliferation [88], dendritic spine development/plasticity [89], and myelin formation [90]. In a similar manner, we propose that this steroid plays a maturational role by signaling the permanent removal of the EGL once its job of producing new neurons is complete [19,20]. As a result, this developmental toxicity can be viewed within the larger context of transitioning the body to a more mature state with the signaling of EGL apoptosis being one of several effects. For the full-term infant, all of these GC initiated events work synergistically to prepare the body for the next stage of life. However, for the prematurely born infant, these events can be at odds with each other. In particular, GC induced maturation of the lungs is needed for appropriate respiration yet the EGL has not finished producing new neurons in the cerebellum. As a result, GC therapy leads to the paradoxical improvement in respiratory function yet precociously eliminates the EGL thereby stunting cerebellar size. While this may seem to propose a dilemma for the clinician, it may be possible to minimize GC’s harmful effects by taking advantage of the natural protection afforded by the HSD2 enzyme. This would involve using hydrocortisone (or other GCs HSD2 can metabolize) rather than the commonly used synthetic GCs (such as dexamethasone and betamethasone) which are poor substrates for this same enzyme [88]. 
